# Case Report: Severe gastric atrophy associated with a novel homozygous variant in *LRBA*

**DOI:** 10.3389/fped.2025.1649994

**Published:** 2025-11-25

**Authors:** Shilin Yuan, Ting Li, Yu Tong, Zhiling Wang

**Affiliations:** 1 Key Laboratory of Birth Defects and Related Diseases of Women and Children, Ministry of Education, Department of Gastroenterology, West China Second University Hospital, Sichuan University, Chengdu, Sichuan, China; 2 Key Laboratory of Birth Defects and Related Diseases of Women and Children, Ministry of Education, Sichuan University, Chengdu, Sichuan, China

**Keywords:** severe gastric atrophy, homozygous variant, *LRBA*, immunodeficiency, case report

## Abstract

**Background:**

The prevalence of atrophic gastritis is challenging to determine, considering that it is typically asymptomatic and that its definition and diagnosis vary among studies. Furthermore, gastric atrophy is underreported and is often considered an underdiagnosed condition in pediatrics.

**Case summary:**

This report describes the case of a 13-year-old girl who initially presented with autoimmune hemolytic anemia, followed by extensive severe gastric mucosal atrophy and erosions with severe growth retardation inconsistent with autoimmune gastritis (AIG). To our knowledge, this represents the first documented case of such an extensive and severe atrophic gastritis identified in Sichuan Province, China. Genetic analysis revealed that the patient exhibited a homozygous mutation in c.229_230del; p.Gln77Valfs*2:NM_001364905.1 in the gene encoding the lipopolysaccharide-responsive beige-like anchor protein (*LRBA*). This mutation led to the loss of normal protein function via nonsense-mediated decay of mRNA or premature termination of the encoded amino acid sequence. This case elucidates a distinct and severe phenotype form of atrophic gastritis caused by the c.229_230del; p.Gln77Valfs*2 homozygous mutation in *LRBA*, expanding the clinical spectrum of *LRBA* deficiency.

**Conclusion:**

Our findings may provide insight into the study of the mechanism of atrophic gastritis and the diagnosis and treatment of severe forms of this condition in pediatric patients.

## Introduction

Atrophic gastritis is defined as the loss of gastric glands, with or without metaplasia, under chronic inflammation conditions mainly caused by *Helicobacter pylori* (*H. pylori*) infection or autoimmunity (1). The prevalence of atrophic gastritis is difficult to establish owing to its typically asymptomatic nature and a lack of consensus regarding its definition and diagnosis in the literature (2, 3). The incidence rate of atrophic gastritis has been reported to range from 0% to 11% per year, and approximately 1% of the patients are not infected with *H. pylori (*3). Atrophic gastritis in pediatric patients is rare, with an incidence rate of 0.15% (4) and it has not yet been well characterized (5). Lipopolysaccharide-responsive beige-like anchor (*LRBA*) protein deficiency is a primary immunodeficiency disease with a prevalence of <1 per million (6). Approximately 14% of the patients with *LRBA* deficiency develop atrophic gastritis (7), as characterized by recurrent vomiting with weight loss and anemia (8). This report is the first to describe a unique form of atrophic gastritis caused by the c.229_230del; p.Gln77Valfs*2:NM_001364905.1 homozygous mutation in *LRBA*.

## Case presentation

A 13-year-old girl was admitted to our hospital presenting with “anemia for 9 years, abdominal pain for 2 years, hematemesis and melena for 4 months, and symptom recurrence for 3 days.” The patient presented with anemia and recurrent respiratory infections 9 years ago but did not receive regular treatment or follow-up. Intermittent upper abdominal pain appeared 2 years prior, and hematemesis and melena occurred 4 months prior. During the course of the disease, the patient received therapeutic interventions such as methylprednisolone, gamma globulin, acid-suppressive therapy, and red blood cell transfusion. The symptoms were initially ameliorated with treatment. However, following self-discontinuation of corticosteroid therapy, the symptoms recurred, necessitating the re-initiation of oral methylprednisolone and acid-suppressant drugs. Three days before admission, the patient was admitted again with upper abdominal pain accompanied by hematemesis and melena without obvious cause. The patient gradually stopped gaining weight and growing in height at the age of 10. The patient was born to consanguineous parents.

**Figure 1:**
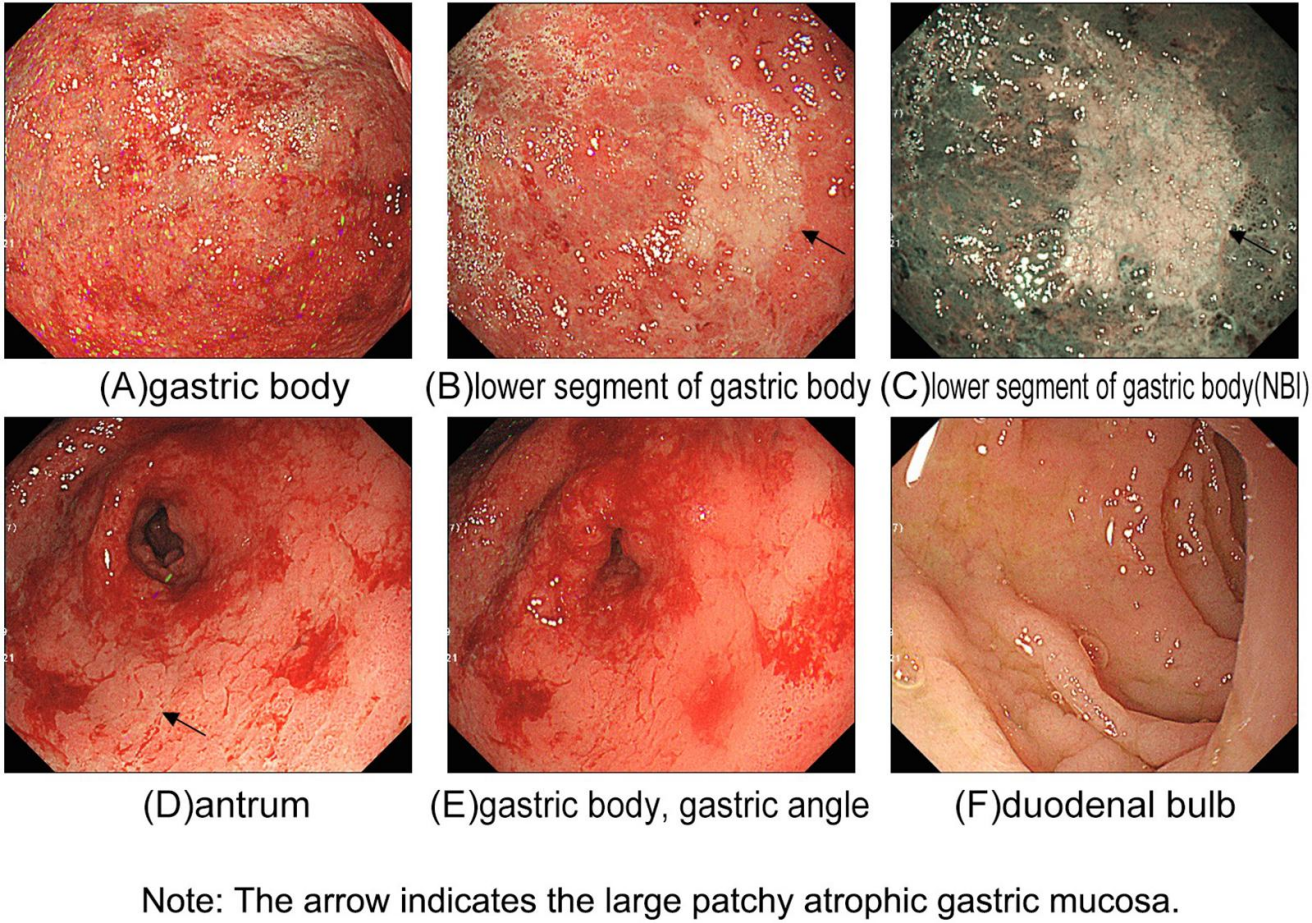
Upper gastrointestinal endoscopy examination of the patient.

**Figure 2:**
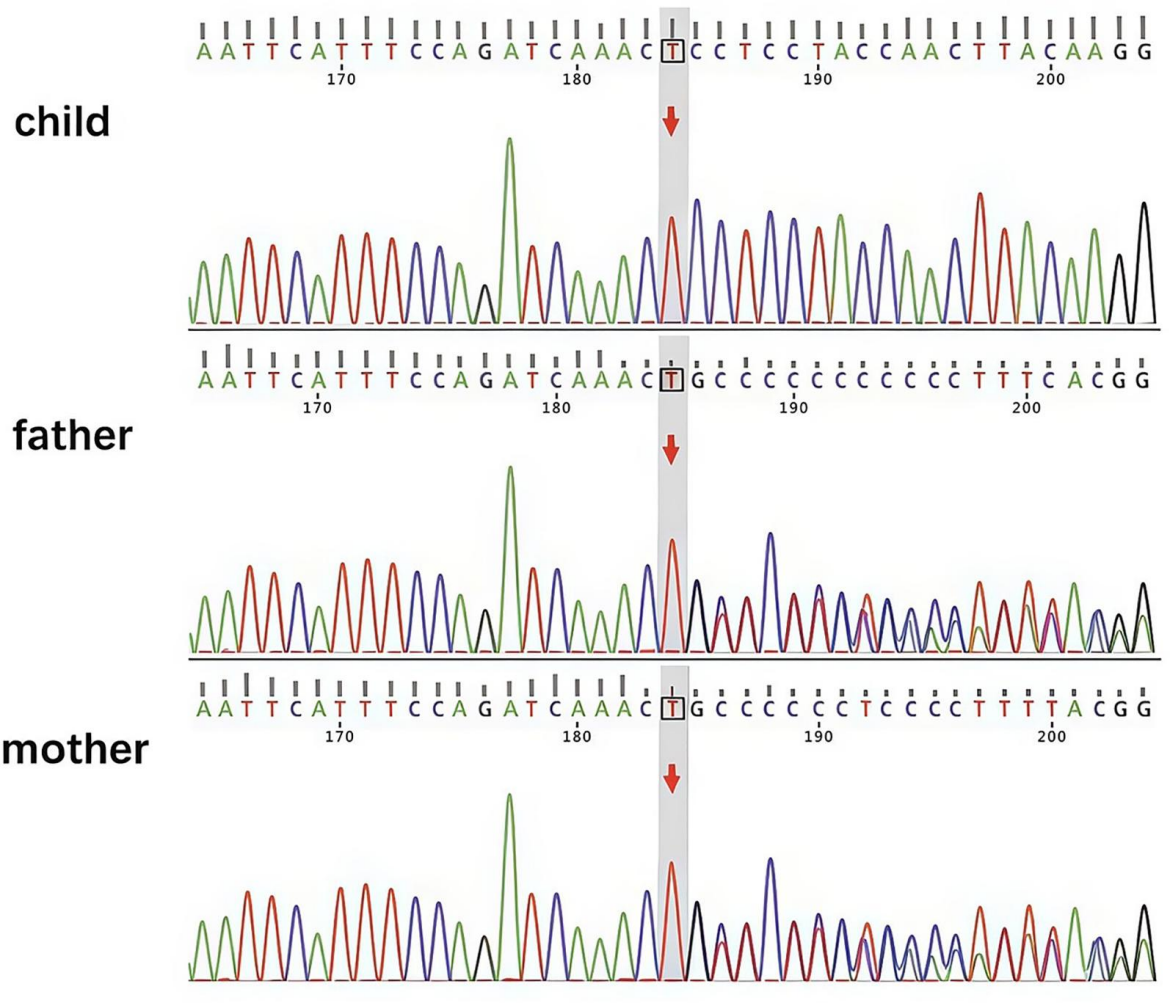
Sanger sequencing chromatogram showing the homozygous mutation c.229230del; p.Gln77Valfs*2:NM001364905.1 in LRBA of the patient, reproduced from a diagnostic test report provided by KingMed Diagnostics with permission.

Based on her physical examinations at the time of admission, her weight was 21.5 kg and her height was 121 cm. Laboratory tests upon admission revealed the following: white blood cell count 2.6 × 10^9/L; neutrophil percentage 9.6%; red blood cell count 4.06 × 10^12/L; mean corpuscular volume 62.8 fL; mean corpuscular hemoglobin concentration 263.0 g/L; hemoglobin 67 g/L; reticulocyte percentage 2.72%; platelet count 164 × 10^9/L; a positive direct antihuman globulin test. Bone marrow examination revealed microcytic hypochromic anemia with granulocytic and megakaryocytic hyperplasia and no abnormal cells. Upper gastrointestinal endoscopy revealed chronic atrophic gastritis with extensive erosions (Figure 1). Pathological assessment further indicated chronic active atrophic gastritis with severe, diffuse atrophy of the mucosal epithelium and intrinsic glandular tissue in the gastric body, antrum, and angle. *H. pylori* infection was negative. Capsule endoscopy suggested villous atrophy throughout the small intestine with fissures and mosaic sign alterations on the mucosal surface, indicating a high possibility of autoimmune enteritis. Immunological profiling demonstrated decreased levels of T lymphocytes, CD4^+^T lymphocytes, and immunoglobulins signified a reduction in both cellular and humoral immunity (Tables 1, 2). Parietal cell and intrinsic factor antibodies were negative. Whole-exome sequencing results showed that the patient had a homozygous mutation c.229_230del; p.Gln77Valfs*2:NM_001364905.1 in *LRBA*, which led to the loss of normal protein function via nonsense-mediated decay of mRNA or premature termination of the encoded amino acid sequence. The analysis of next-generation sequencing data revealed that the patient had inherited the mutation from her parents, confirming that both parents were heterozygous carriers of this mutation (Figure 2).

**Table 1:** Immune-related tests in *LRBA* of the patient: cellular immunity test.

No.	Test item	result	Unit	Reference value
1	T lymphocytes percentage (CD3+%)	67.8	%	52–78
2	T lymphocyte count (CD3+)*	0.70↓	10^9/L	0.80–3.50
3	Helper T lymphocytes percentage (CD3+CD4+%)	28.4	%	25–48
4	Helper T cell count (CD3+CD4+)*	0.29↓	10^9/L	0.40–2.10
5	inhibitory T lymphocytes percentage (CD3+CD8+%)	32.1	%	9–35
6	Inhibitory T lymphocyte count (CD3+CD8+)	0.33	10^9/L	0.20–1.20
7	CD3+CD4+/CD3+CD8+ (CD4/CD8)*	0.9↓	-	1.5–2.0
8	B lymphocyte percentage (CD19+%)*	24.5↑	%	8–24
9	B lymphocyte count (CD19+)	0.25	10–9/L	0.20–0.60
10	NK lymphocytes percentage (CD3-CD16+56+%)	7.5	%	6–27
11	NK lymphocytes count (CD3-CD16+56+)*	0.08	10^9/L	0.07–1.20

**Table 2:** Immune-related tests in *LRBA* of the patient: humoral immunity test.

No.	Test item	Result	Reference value	Unit
1	IgG*	3.33↓	5.29–21.9	g/L
2	IgA	0.49	0.41–3.95	g/L
3	IgM*	0.41↓	0.48–2.26	g/L
4	C3*	0.38↓	0.70–2.06	g/L
5	C4*	0.08↓	0.11–0.61	g/L
6	C1q	16.55	15.7–23.7	mg/mL
7	IgE	<17.00	200	IU/mL

After admission, the patient was treated with oral methylprednisolone, intravenous immunoglobulin, proton-pump inhibitor (starting with omeprazole and then transitioning to esomeprazole), total parenteral nutrition, and fasting, among other medications (Table 3). Gastrointestinal symptoms or severe respiratory infections did not recur for more than 10 months after discharge. At the 13-month post-discharge follow-up, the hemoglobin level was 80 g/L and demonstrated a declining trend. Treatment with sirolimus/abatacept or allogeneic hematopoietic stem cell transplantation (HSCT) was recommended but deferred by the patient due to financial constraints. Currently, the patient continues to take methylprednisolone tablets (8 mg, bid) as oral maintenance treatment.

**Table 3:** The condition and corresponding treatment timeline of the case patient.

Timeline	Symptoms	Treatments
Prior to 2023.9.27	Anemia, recurrent respiratory tract infections, abdominal pain, hematemesis and melena	Without regular treatment and follow-up
2023.9.27 (Initial admission)	Upper abdominal pain, hematemesis, and black stool	Methylprednisolone 12 mg qd##, omeprazole 20 mg qd, and immunoglobulin 20 g once#
2023.10.3	Persistent abdominal pain and repeated vomiting with hematemesis	Fasting, total parenteral nutrition, methylprednisolone 20 mg bid##, omeprazole 25 mg qd, and immunoglobulin 20 g once#
2023.10.31	No complaint of abdominal pain, no hematemesis, and no hematemesis	Methylprednisolone tablet 18 mg in the morning and 16 mg in the afternoon, with a weekly reduction of 2 mg, and esomeprazole 20 mg qd
2025.1.10	Anemia, hemoglobin level of 80 g/L	Sirolimus/Abatacept or HSCT recommended declined by the patient

# Did not receive regular immunoglobulin replacement therapy,

## qd: once daily, bid: twice daily.

## Discussion

Gastric atrophy is a complex syndrome that is a well-established entity in adults. Nevertheless, it remains rarely described and is even considered underdiagnosed in pediatrics (9). Gastric atrophy is most commonly associated with AIG or as a consequence of *H. pylori* infection. AIG is a chronic inflammatory process characterized by the destruction of oxyntic glands and their replacement by atrophic and metaplastic mucosa, accompanied by lymphoplasmacytic infiltration of the lamina propria. However, the severe involvement of the gastric antrum in our case was inconsistent with the typical pathological features of AIG. Furthermore, Parietal cell and intrinsic factor antibodies were both negative, and the anemia was not megaloblastic. Therefore, AIG was ruled out. In addition, the patient have no evidence of *H. pylori* infection. Consequently, the findings in this case suggest that other types of atrophic gastritis may exist independently of the recognized ones.

The patient had a homozygous mutation c.229_230del; p.Gln77Valfs*2:NM_001364905.1 in *LRBA*, which is located at 4q31.3, contains 57 exons, and encodes a protein containing 2,851 amino acids (10). We performed a pathogenicity assessment of this variant according to the American College of Medical Genetics and Genomics (ACMG) guidelines. This deletion creates a frameshift and premature termination codon. Multiple lines of evidence support the pathogenicity of this variant: (1) It is a loss-of-function variant, and loss of function of the *LRBA* gene is a known disease mechanism (PVS1); (2) The allele frequency of this variant is absent from population genomic databases such as gnomAD and ClinVar (PM2); (3) The patient's clinical phenotype (severe immune dysregulation and gastrointestinal pathology) is highly specific for *LRBA* deficiency (PP4); (4) Genetic testing confirmed the homozygous status of this variant, consistent with an autosomal recessive inheritance pattern (PM3). These findings indicate that the p.Gln77Valfs*2:NM_001364905.1 variant is pathogenic. Research has shown that the *LRBA* protein is involved in the vesicular transport of immune effector molecules such as cytotoxic T lymphocyte-associated antigen-4, which plays a critical role in controlling T-cell activation and tolerance by mediating the immunosuppressive function of regulatory T cells (11). Therefore, *LRBA* deficiency can cause a range of immune dysregulations. The International Union of Immunological Societies has now classified *LRBA* deficiency as a T-cell deficiency with immune dysregulation (12).

*LRBA* deficiency is a primary immunodeficiency disease characterized by considerable heterogeneity in immunological and clinical phenotypes. As the largest immune organ in the human body, the gastrointestinal tract is one of the most commonly affected organs in this disease. Consequently, patients may manifest gastrointestinal pathology such as atrophic gastritis and enteropathy. Approximately 62% of patients with *LRBA* deficiency developed enteropathy. Although our patient exhibited villous atrophy throughout the small intestine with fissures and mosaic sign alterations in the mucosa, the stomach was more severely affected. She had chronic atrophic gastritis with extensive erosion and severe atrophy of the diffuse mucosal epithelium and the intrinsic glandular tissues. After excluding *H. pylori* infection and classic autoimmune gastritis, we propose that the severe global gastric atrophy arises from a unique immunopathological mechanism triggered by *LRBA* deficiency. It is known that *LRBA* deficiency disrupts CTLA-4 homeostasis, leading to impaired Treg cell function and aberrant activation of effector T cells. Based on this, we propose a reasonable hypothesis: the complete loss of *LRBA* protein function in our patient may have provoked a potent, T-cell-dominated autoimmune attack targeting the gastric mucosa. This cell-mediated immune response, independent of traditional autoantibodies, resulted in widespread glandular destruction, thereby explaining why the pattern of atrophy differs from both *H. pylori*-associated gastritis and classic autoimmune gastritis. This mechanistic hypothesis warrants future validation, for instance, phenotypic analysis of lymphocytes infiltrating the patient's gastric mucosa. This is the first pediatric case of such an extensive and severe atrophic gastritis identified in Sichuan Province, China. Moreover, this is the first report of such a unique form of atrophic gastritis, The significance of our case lies in providing crucial human clinical evidence for elucidating this novel immune-mediated pathogenic pathway in atrophic gastritis.

Currently, no standard treatment exists for *LRBA* deficiency. Conventionally, most patients benefit from symptomatic therapies such as anti-infective, immunosupportive, and/or immunosuppressive treatments for immune dysregulation. In the present case, gastrointestinal symptoms and respiratory infections were initially controlled with such a regimen. However, follow-up revealed a progressive decline in hemoglobin levels, indicating that maintenance treatment with glucocorticoids failed to provide sustained disease control in the long term. Importantly, the therapeutic landscape for *LRBA* deficiency is evolving beyond symptomatic care. Targeted immunomodulatory agents, such as sirolimus [a mechanistic target of rapamycin (mTOR) inhibitor that curbs aberrant T-cell activation] and abatacept (a CTLA-4-Ig fusion protein that directly compensates for the CTLA-4 trafficking defect caused by *LRBA* deficiency), have shown promising results in recent years. These therapies are associated with improved survival rates and reduced disease burden by more precisely addressing the underlying immunopathology. They can serve either as long-term management options or as a bridge to a potentially curative treatment (13–15). Allogeneic hematopoietic stem cell transplantation (HSCT) remains the only definitive curative option for eligible patients with severe disease (16, 17). For this particular patient, we recommended a treatment escalation to either sirolimus/abatacept or HSCT. However, due to socioeconomic constraints and familial preferences, the family has thus far opted to continue with maintenance glucocorticoids alone. This case highlights not only the clinical challenge of severe *LRBA* deficiency but also the barriers to accessing advanced, targeted therapies. Our experience underscores the urgent need to develop and disseminate standardized management guidelines while addressing the practical hurdles in implementing precision medicine for rare diseases.

In conclusion, our report describes a unique and severe phenotype of pediatric global gastric atrophy driven by a novel homozygous *LRBA* mutation. This case provides two critical insights: first, it highlights the critical role of the *LRBA*-CTLA-4 axis and T-cell-mediated immunity in the pathogenesis of a severe, atypical form of atrophic gastritis, offering a new direction for mechanistic research. Second, it suggests that for severe pediatric atrophic gastritis with an unknown etiology, establishing a clinical phenotype-guided diagnostic pathway that incorporates early genetic testing and ultimately directs targeted therapy may provide new strategies for the diagnosis and treatment of such diseases.

## Data Availability

The datasets presented in this study can be found in online repositories. The names of the repository/repositories and accession number(s) can be found in the article/Supplementary Material.
