# Correction: Case Report: Severe gastric atrophy associated with a novel homozygous variant in *LRBA*

**DOI:** 10.3389/fped.2026.1760475

**Published:** 2026-02-04

**Authors:** Shilin Yuan, Ting Li, Yu Tong, Zhiling Wang

**Affiliations:** 1 Key Laboratory of Birth Defects and Related Diseases of Women and Children, Ministry of Education, Department of Gastroenterology, West China Second University Hospital, Sichuan University, Chengdu, Sichuan, China; 2 Key Laboratory of Birth Defects and Related Diseases of Women an Children, Ministry of Education, Sichuan University, Chengdu, Sichuan, China

**Keywords:** severe gastric atrophy, homozygous variant, *LRBA*, immunodeficiency, case report

**Corresponding author**

In the published article, one of the authors was erroneously omitted of being a corresponding author.

Author Zhiling Wang was erroneously omitted as a corresponding author. The original version of this article has been updated.

